# Entecavir-Associated Thrombocytopenia: A Case Report and Review of the Pathophysiology, Diagnosis, and Treatment of a Rare but Reversible Cause of Thrombocytopenia

**DOI:** 10.1155/2019/4319148

**Published:** 2019-03-12

**Authors:** Binoy Yohannan, Dai Chu N. Luu, Mark Feldman

**Affiliations:** ^1^Department of Internal Medicine, Texas Health Presbyterian Hospital, Dallas, Texas 75231, USA; ^2^Department of Internal Medicine, Division of Hematology and Oncology, Texas Health Presbyterian Hospital, Dallas, Texas 75231, USA

## Abstract

Drug-associated thrombocytopenia is often unrecognized. We report a 76-year-old female with lymphoma who presented with easy bruising and oral bleeding. She had undergone screening for hepatitis B virus (HBV) prior to starting rituximab and was found to have hepatitis B core serum antibody (IgG anti-HBc). She was therefore treated with prophylactic entecavir 0.5 mg daily to prevent reactivation of HBV. Her initial platelet count was 136,000/mm^3^. Five days after starting entecavir, she presented with bruising and oral bleeding and was found to have a platelet count of 7,000/mm^3^. A coagulation profile and the rest of the blood parameters (RBC and WBC counts) were normal. Entecavir was stopped, and she was given 3 units of apheresed platelets followed by intravenous immunoglobulin (1 g/kg) for 5 consecutive days. Her platelet counts improved and normalized in one week. She was diagnosed with entecavir-induced thrombocytopenia based on the temporal relationship and after carefully excluding alternate causes of thrombocytopenia. This case highlights the importance of recognizing drug-induced thrombocytopenia (DITP) as a reversible cause of thrombocytopenia.

## 1. Introduction

The most common side effects of entecavir therapy for hepatitis B virus (HBV) include headache, fatigue, dizziness, nausea, and vomiting. Very rarely, patients can develop lactic acidosis and hepatic steatosis. Although other nucleoside analogues with activity against HBV such as lamivudine [1] and adefovir [2] have been associated with drug-induced thrombocytopenia, the incidence is very low with entecavir. Here, we report a case of an elderly female with serological evidence of past HBV infection who developed severe thrombocytopenia with bleeding after she had received entecavir for 5 days. This is the second case report of entecavir-induced thrombocytopenia.

## 2. Case Presentation

A 76-year-old female presented with a 2-day history of easy bruising and bleeding from her mouth. She denied epistaxis, melena, hematochezia, hematuria, or other bleeding manifestations. She had estrogen receptor positive stage 1 breast cancer 24 years ago for which she underwent lumpectomy followed by tamoxifen for 5 years. She has had regular mammograms since then, and there has been no evidence of local disease recurrence. Fourteen years ago, she was diagnosed with Waldenstrom's macroglobulinemia for which she was treated with chlorambucil and prednisone for 3 months with significant improvement. Her plasma cell dyscrasia has been quiescent. M-spike on serum protein electrophoresis was minimal. She had intermittent hospitalizations for pneumonia, thought to be related to functional immunoglobulin deficiency. She was on intravenous immunoglobulin replacement for 6 months, but it was discontinued later. Three years ago, she presented with pleural and pericardial effusions, and pleural biopsy showed activated B-cell (ABC) diffuse large B-cell lymphoma (DLBCL). A PET scan showed uptake in the pleural space with no lymphadenopathy. A bone marrow biopsy showed no evidence of DLBCL, and she was treated with rituximab and bendamustine for 3 months and remained in remission for the next three years. Four weeks prior to the current admission, she presented with fatigue, night sweats, and 10-pound weight loss and was found to have retroperitoneal lymphadenopathy. She underwent CT-guided core biopsy of the retroperitoneal nodes that confirmed recurrence of her DLBCL. A repeat bone marrow biopsy showed normocellular bone marrow with progressive trilineage hematopoiesis, but no evidence of DLBCL. She was scheduled to start salvage therapy for DLBCL with rituximab and lenalidomide. Viral hepatitis serologies were performed prior to initiation of rituximab, showing evidence of prior hepatitis B infection (IgG anti-HBc antibody). She was therefore started on prophylactic entecavir 0.5 mg daily 5 days prior to the current admission. Other medical problems included diabetes mellitus, hypertension, atrial fibrillation, gout, hypothyroidism, hyperlipidemia, and osteoporosis. Her other medications besides entecavir included allopurinol, amiodarone, rosuvastatin, diltiazem, pantoprazole, dabigatran, ramipril, and levothyroxine. No recent change in these medications had occurred.

**Figure 1:**
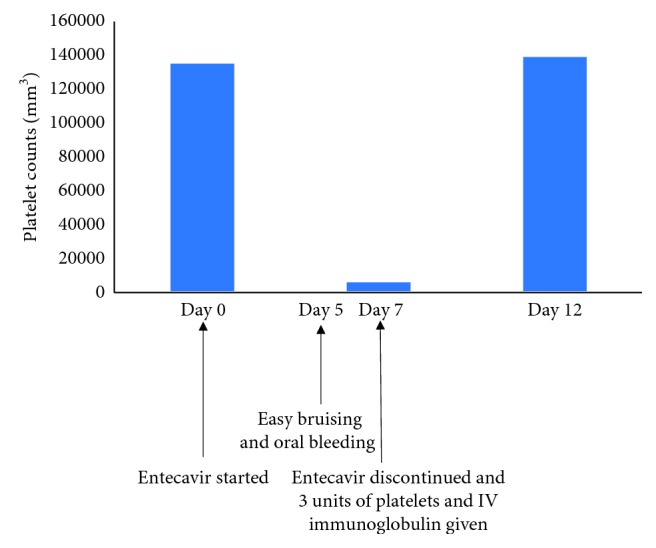
Course of drug-induced immune thrombocytopenia in a patient treated with entecavir. Entecavir was prescribed for prophylaxis to prevent HBV reactivation before initiating rituximab therapy. Starting from day 5 of treatment, the patient reported having easy bruising and bleeding from the oral cavity. She came to the office on day 7, and her platelet count was 7000 per cubic millimeter. Entecavir was discontinued immediately, and 3 units of apheresed platelets and IV immunoglobulin were given (days 7–11). The platelet counts improved and normalized in a week.

On physical examination, her temperature was 98.3°F with blood pressure 100/68 mmHg, pulse 90 beats per min, and respiratory rate 18 per minute. She looked tired but had no pallor, icterus, or generalized lymphadenopathy. The anterior nares were clear without epistaxis. Mucous membranes were moist, and the oropharynx revealed two scabs in the buccal mucosa with dried blood. She had normal S1 and S2 without any rubs, murmurs or gallops, and normal vesicular breath sounds. Her abdomen was soft and nontender without hepatosplenomegaly or ascites. Skin was warm and dry without petechia, purpura, or ecchymosis. The rest of the physical exam was unremarkable. Complete blood count showed thrombocytopenia with a platelet count of 7000/mm^3^. Peripheral blood smear showed no evidence of platelet clumping. The hemoglobin and white blood cell counts were normal. Thyroid function tests and a coagulation profile were essentially normal. Her last recorded platelet count 10 days prior to starting entecavir was 136,000/mm^3^. Her baseline platelet count range is between 120,000 and 150,000/mm^3^. Entecavir was discontinued, but her other drugs were continued. She received 3 units of platelets followed by intravenous immunoglobulin (1 g/kg) for 5 consecutive days. Her platelet counts improved and normalized in one week (Figure 1). She then received lenalidomide and rituximab for her DLBCL, but did not receive any HBV prophylaxis as it was considered unsafe to expose her to any of the other nucleoside analogues.

## 3. Discussion

Patients with chronic hepatitis B or serological evidence of past infection are at risk for viral reactivation when treated with immunosuppressive therapy. B-cell depleting agents such as rituximab have the highest risk of HBV reactivation [3, 4]. The nucleos(t)ide analogues such as entecavir (ETV) and tenofovir can be used for HBV prophylactic therapy, and it should be continued for at least 12 months for those receiving B-cell depleting agents [5]. ETV, a guanine nucleoside analogue, has been associated with thrombocytopenia in only one case report in which an elderly female with hepatitis B cirrhosis suffered from severe thrombocytopenia after she received ETV therapy; and this was successfully treated by discontinuation of entecavir and intravenous immunoglobulin [6]. Clinicians should consider drug-induced thrombocytopenia (DITP) in the differential diagnosis of a patient on ETV therapy presenting with unexplained isolated thrombocytopenia. DITP can mimic primary immune thrombocytopenia (ITP); however, differentiating these syndromes is important to avoid unnecessary immunosuppressive treatments and to avoid future exposure to the sensitizing drug. Hence, it is important for clinicians to be aware of this adverse drug event and have a general understanding of the common drugs associated with thrombocytopenia. Drugs commonly implicated as triggers of DITP include heparin, quinine, glycoprotein II_b_ III_a_ inhibitors, gold salts, antibiotics (e.g., linezolid, rifampin, sulfonamides, and vancomycin), antiepileptics (e.g., carbamazepine, phenytoin, and valproic acid), analgesics (e.g., acetaminophen, diclofenac, and naproxen), diuretics (e.g., hydrochlorothiazide and chlorothiazide), and chemotherapeutic agents including proteasome inhibitors [7, 8]. DITP can be either nonimmune or immune-mediated. Nonimmune DITP can be secondary to bone marrow suppression (e.g., cytotoxic chemotherapy and linezolid) or inhibition of proplatelet formation in megakaryocytes (proteasome inhibitors) [9]. Immune-mediated thrombocytopenia in the presence of certain drugs can be explained by the following mechanisms: (a) classic drug-dependent platelet antibodies (quinine-type); (b) hapten-induced platelet antibodies (e.g., penicillin); (c) fiban-dependent antibodies (e.g., tirofiban); (d) fragments antigen binding monoclonal antibodies (e.g., abciximab); and (e) drug-induced autoantibody formation (e.g., gold). Drug-induced autoantibodies can either attach firmly to the epitopes on the platelet surface and cause accelerated destruction or target the megakaryocyte causing immune-mediated suppression of platelet production [10–12]. These autoantibodies can also affect the other cell lines including neutrophils and red blood cells causing neutropenia and immune hemolytic anemia, respectively [13].

Clinicians should elicit a detailed drug exposure history to establish the diagnosis of DITP, specifically enquiring about prescription drugs, over the counterdrugs, herbal preparations, certain foods, and beverages [7]. There is no definitive diagnostic test for DITP, but instead DITP is usually diagnosed by the temporal association of exposure to a suspected drug with an acute, severe thrombocytopenia, with the nadir platelet counts often less than 20,000/mm^3^ [11, 14]. Patients are often exposed to the sensitizing drug for about 5–7 days, but DITP can also occur following intermittent use of a drug over a long time period. Glycoprotein II_b_ III_a_ inhibitors such as abciximab can cause severe thrombocytopenia within 1 or 2 days, even with the first exposure to the drug [15]. George et al. [8] proposed 4 clinical criteria and level of evidence to help establish the likelihood that a specific drug is responsible for thrombocytopenia (Table 1). Our patient met 3 of these criteria and thus probably had DITP. Furthermore, she had had a bone marrow biopsy ten days prior to the presentation, the megakaryocyte morphology was normal, and she had no evidence of malignant marrow infiltration. Autoimmune thrombocytopenia is a common hematologic complication of B-cell lymphomas and could potentially confound thrombocytopenia in this patient, but the temporal association with entecavir exposure and quick resolution after discontinuation of the drug favors DITP. Except for heparin, testing for drug-induced autoantibodies is not widely available and hence not helpful in clinical decision-making. Drug metabolites produced in vivo can act as a sensitizing agent, and thus, even in cases of typical DITP, antibody testing against the parent drug can be negative [16]. If the clinical suspicion is strong for DITP, reexposure to the suspected drug can be considered to document drug sensitivity. However, in sensitized individuals, a conventional dose can cause severe thrombocytopenia and bleeding. Therefore, if rechallenge is considered, one should start with a very low dose and closely monitor platelet counts and clinical status [17]. In cases of DITP, once the offending drug is discontinued, symptoms usually resolve quickly, and the platelet count returns to normal in less than a week. Prolonged thrombocytopenia after discontinuation of the suspected drug is evidence against a causal role for that drug.

The clinical presentation of DITP can vary from asymptomatic thrombocytopenia to life-threatening hemorrhage. Patients may have epistaxis, gum bleeding, oral mucous membrane blood blisters, melena, hematochezia, and hematuria when the platelet count drops below 20,000/mm^3^, and deaths from bleeding have been reported [14]. Most patients with mild to moderate DITP can be managed conservatively with discontinuation of the offending drugs and closely monitoring the platelet count. When there is uncertainty about the causative drug, all medications should be discontinued [7]. Patients with severe thrombocytopenia and bleeding manifestations are at high risk for life-threatening hemorrhage and should be managed aggressively with platelet transfusions [12]. The role of glucocorticoids in patients with DITP is controversial, and there is no evidence that it improves clinical outcomes. Nevertheless, glucocorticoids are often used in unexplained isolated thrombocytopenia, since ITP cannot be easily excluded. When DITP is strongly suspected, it is appropriate to stop glucocorticoid therapy abruptly after the platelet count returns to normal. In DITP, plasma exchange [18] and intravenous immune globulins [19] have been used in patients with severe thrombocytopenia and bleeding manifestations, but the benefit of these treatments is uncertain [12].

Once drug sensitivity is documented, autoantibodies persist for life, and patients should be advised to avoid the drug in future, and this should be documented in their medical records as a serious “allergy.”

## 4. Conclusion

Entecavir-induced thrombocytopenia is a reversible cause of thrombocytopenia potentially mediated via numerous immune and nonimmune mechanisms. Clinicians should maintain a high index of clinical suspicion for DITP in the patient presenting with severe thrombocytopenia soon after starting entecavir and quick resolution after discontinuing the drug. Early recognition and prompt discontinuation of entecavir is critical to avoid life-threatening hemorrhage.

## Figures and Tables

**Table 1:** Clinical criteria and levels of evidence for evaluation of patients with suspected drug-induced thrombocytopenia.

*Clinical criteria*
(1) Drug administration preceded thrombocytopenia; complete and sustained recovery from thrombocytopenia is noted after drug discontinuation
(2) Other drugs administered prior to thrombocytopenia were continued or reintroduced after discontinuation of the suspected drug
(3) Other etiologies of thrombocytopenia excluded
(4) Reexposure to the drug resulted in recurrent thrombocytopenia

*Levels of evidence*
(1) Definite: all 4 criteria met
(2) Probable: criteria 1–3 met
(3) Possible: criterion 1 met
(4) Unlikely: criterion 1 not met

Adapted from http://www.ouhsc.edu/platelets and Reference [6].
